# Improving the delivery of care for patients with diabetes through understanding optimised team work and organisation in primary care

**DOI:** 10.1186/1748-5908-4-22

**Published:** 2009-04-27

**Authors:** Martin P Eccles, Gillian Hawthorne, Marie Johnston, Margaret Hunter, Nick Steen, Jill Francis, Susan Hrisos, Marko Elovainio, Jeremy M Grimshaw

**Affiliations:** 1 Institute of Health and Society, University of Newcastle upon Tyne, 21 Claremont Place, Newcastle upon Tyne, NE2 4AA, UK; 2 Newcastle Diabetes Centre, Newcastle General Hospital, Westgate Road, Newcastle upon Tyne, NE4 6BE, UK; 3 College of Life Sciences and Medicine, University of Aberdeen, Polwarth Building, Foresterhill, Aberdeen, AB25 2ZD, UK; 4 Health Services Research Unit, University of Aberdeen, Health Sciences Building, Foresterhill, Aberdeen, AB25 2ZD, UK; 5 National Institute for Health and Welfare, Mannerheimintie 166, Helsinki, Finland; 6 Ottawa Health Research Institute, 1053 Carling Avenue, Room 2-017, Admin Building, Ottawa, ON K1Y 4E9, Canada; 7 Department of Medicine, University of Ottawa, Ontario, Canada, K1H 8M5

## Abstract

**Background:**

Type 2 diabetes is an increasingly prevalent chronic illness and is an important cause of avoidable mortality. Patients are managed by the integrated activities of clinical and non-clinical members of the primary care team. Studies of the quality of care for patients with diabetes suggest less than optimum care in a number of areas.

**Aim:**

The aim of this study is to improve the quality of care for patients with diabetes cared for in primary care in the UK by identifying individual, team, and organisational factors that predict the implementation of best practice.

**Design:**

Participants will be clinical and non-clinical staff within 100 general practices sampled from practices who are members of the MRC General Practice Research Framework. Self-completion questionnaires will be developed to measure the attributes of individual health care professionals, primary care teams (including both clinical and non-clinical staff), and their organisation in primary care. Questionnaires will be administered using postal survey methods. A range of validated theories will be used as a framework for the questionnaire instruments. Data relating to a range of dimensions of the organisational structure of primary care will be collected via a telephone interview at each practice using a structured interview schedule. We will also collect data relating to the processes of care, markers of biochemical control, and relevant indicator scores from the quality and outcomes framework (QOF). Process data (as a proxy indicator of clinical behaviours) will be collected from practice databases and via a postal questionnaire survey of a random selection of patients from each practice. Levels of biochemical control will be extracted from practice databases. A series of analyses will be conducted to relate the individual, team, and organisational data to the process, control, and QOF data to identify configurations associated with high quality care.

**Study registration:**

UKCRN ref:DRN120 (ICPD)

## Background

In the UK, Type 2 diabetes is an increasingly prevalent chronic illness (prevalence now over 3% [1], equating to approximately 50 patients per full time general practitioner). It is an important cause of avoidable mortality. Patients are managed by the integrated activities of clinical and non-clinical members of the primary care team. There are National Institute for Health and Clinical Effectiveness (NICE) guidelines defining standards of care, but studies of the quality of care for patients with diabetes suggest less than optimum care in a number of areas [2]. Quality and outcomes framework (QOF) data for 2004 and 2005 suggest high rates of measurement of clinical and biochemical parameters but lower rates of acting on the results. Only 67% of practices achieved the target of 50% of patients having their hemoglobin A1c (HbA1c) within the target range; the similar figure for blood pressure (BP) (target of 55%) was 70%. Some of this variability will reflect patient physiology/behaviour, but it will also reflect variable clinical management behaviours. General practices, although relatively small organisations, have become more complex in terms of their structures and functions. Few studies have examined factors underlying the organisation and delivery of care in general practice, and only one UK study related them to clinical data [3] demonstrating that team climate, booking interval, and practice size together explained 31% of the variance in diabetes management. These authors, along with others [4,5], identified the need for a better understanding of the relationship between the quality of patient care and individual and organisational factors.

A consistent finding in health services research is that the transfer of research findings into practice is unpredictable and can be a slow and haphazard process [6]. Studies in the USA and the Netherlands suggest that 30 to 40% of patients do not receive care according to current scientific evidence, and 20 to 25% of care provided is not needed or potentially harmful [7,8]. A review of quality of care studies (including diabetes) from UK primary care concluded that 'in almost all studies the process of care did not reach the standards set out in national guidelines or set by the researchers themselves'[2]. In our recently completed randomized controlled trial (RCT), only 25% of diabetic patients received statins and less than 50% had a foot examination. Recognition of this quality gap has led to increased interest in active implementation strategies and implementation research (the scientific study of methods to promote the systematic uptake of research findings into routine clinical practice) over the past fifteen years [9-11]. It has been demonstrated that interventions can be effective, but less information is provided to guide the choice or optimise the components of complex interventions in practice [12]. The effectiveness of interventions varies across different clinical problems, contexts, and organizations, but studies provided scant theoretical or conceptual rationale for their choice of intervention [13], and only limited descriptions of the interventions and contextual data [14]. We have argued previously [15] that the understanding of potential barriers and enablers to implementation is limited. The challenge for implementation researchers is to develop and evaluate a theory-based approach that will offer a generalisable framework for research and support the choice and development of interventions. Such a framework is also needed for the interpretation of implementation study results.

We have conducted a range of relevant work, including: studies of quality of care in diabetes in primary care [16-18]; pragmatic randomised controlled trials of various quality improvement (QI) strategies, including computerisation of guidelines [19], educational messages attached to test results [20,21], an enhanced area wide diabetes register [22], and outreach visiting [23-25]; studies introducing QI strategies into health service organisations [26,27]; and studies exploring the role of theory in the design and conduct of QI studies [15,28-30]. We are currently conducting a cluster RCT of a QI intervention targeting general practitioners (GPs) caring for patients with diabetes in Newcastle. We have also conducted research in organizational development and behavioural change related to health care organizations in primary and secondary care [31-33].

## Aim

The aim of this study is to improve the quality of care for patients with diabetes cared for in primary care by identifying individual, team, and organisational factors that predict the implementation of best practice.

## Objectives

1. To measure attributes of individual health care professionals (HCPs), teams, and their organisation in primary care.

2. To measure a range of dimensions of organisational structure in primary care.

3. To measure the process of care, markers of biochemical control, and QOF scores.

4. To relate the data from objectives one and two to the data from objective three, and thereby identify configurations associated with high quality care.

## Methods

### Design

The overall design is a predictive study where a series of attributes of individuals, their teams, and organisations are measured, and their ability to predict quality of care over the subsequent 12 months is explored in the analysis.

### Participants and setting

The study will be based in 100 general practices within the UK's MRC general practice research framework (GPRF). There are two major reasons why the use of such a network of practices is necessary. First, in this study it is important to have high-quality data on clinical behaviours. In our previous work, we have used routinely available (prescribing) data as our measure of performance, and we have experienced problems with the specificity of such measures [34]. Second, it is of fundamental importance to this study that we obtain as near as possible complete 'sets' of questionnaires from all relevant members of the primary care team in order to allow the production of robust collective team level values. Although in previous studies we have been successful in obtaining sufficient responses, these have usually been across a large number of practices with only one or two responses per practice. Practices will be recruited by postal invitation with telephone follow-up via the GPRF.

Participants will be all clinical and non-clinical members of the primary care team within each practice. We will collect information about the organisation and team functioning of the primary care team from both clinical and non-clinical team members and information relating to clinical behaviours from clinical team members. Six different aspects of care delivery for patients with diabetes will be examined: glycaemic control, BP control, foot examination, weight management, patient education, and self monitoring. Anonymous clinical records of patients in the study practices will provide the data on clinical variables. A random sample of 100 patients per practice will provide data on clinician behaviours.

### Predictor variables

#### Data relating to practice organisational structure

We will collect details of practice structures and function informed by previous studies [3,5]: practice demographics (including practice list size; training status of the practice, and postcodes covered); routine booking intervals for patient consultations; staffing levels of practice staff (numbers of, and number of sessions worked by, doctors, practice employed nurses, and administrative staff); skill mix (ratio of doctors to non-medical clinical staff, and of clinical to administrative staff); organisation of care for the clinical conditions (including specialisation within the clinicians).

#### Data relating to individual staff's perceptions of the clinical care of their patients with type 2 diabetes, team functioning, and practice organisation

##### Choosing theories

The theoretical frameworks included in this study have been carefully chosen after a process of critical consideration [35]. Multiple theories are required as no one theory covers all of the relevant domains of behaviour [36]. We have chosen theories that predict behaviour change and that have standard methods of operationalisation. Especially in the context of diabetes care, a combination of individual and team/organisational measures has great potential for added understanding.

Theories on human behaviour (and especially adult behaviour change) can be categorised in many different ways. We propose to use theories that cover individuals' cognitions, habitual behaviour, and team performance and decision-making. Individuals' cognitions about the six clinical behaviours will be measured using situation-outcome, outcome and self-efficacy expectancies from social cognitive theory [37], attitude, subjective norms, perceived behavioural control and intention from the theory of planned behaviour (TPB) [38], the self-report habit index [39], and action planning and coping planning [40]. We will also measure self-reported past behaviour.

Based on Bandura's social cognitive theory [37], we will measure three kinds of expectancies – situation-outcome, outcome, and self-efficacy expectancies. TPB proposes that the strength of an individual's intention (or motivation) to engage in a behaviour, and the degree of control they feel they have over that behaviour (perceived behavioural control, or PBC) are the proximal determinants of engaging in it [38]. In turn, intention is influenced by three variables: attitudes towards the behaviour, subjective norms, and PBC. Anticipated regret (an extension to TPB) reflects the notion that the anticipation of regret that would follow the adoption or non-adoption of a behaviour influences its adoption. Including this construct improves the predictive value of TPB [41]. The self-report habit index [39] is a 12-item measure that breaks down the habit construct into a number of features (perceptions of frequency, automaticity, and self-identity). Gollwitzer [42] has identified implementation intentions as explicit plans about when and where a goal intention will be achieved [42]. A relatively new concept in health behaviour research experimental studies suggest that people who have formulated plans are more likely to translate their intentions into action than those who have not [43,44]. The concept has recently been further developed by Sniehotta [40], who proposes the two distinct dimensions of plans: action planning (planning the initiation of a behaviour) and coping planning (planning what to do when barriers to action are encountered in order to maintain changed behaviours).

Individual staff's perceptions of team performance and decision making will be measured through their ratings of: work characteristics, as defined by the demand-control model [45] ('job control'); characteristics of employee interaction, as defined by team climate research [46]; and the characteristics of decision making and managerial procedures, as defined by organisational justice research [47,48].

#### Developing theory-based measures

Using the methods that we have described previously [35], measures will be developed for each predictor variable for the survey. Wherever possible we will use existing measures as a starting point in this process and will follow the standard procedures that have been described to develop measures of these theoretical constructs. Questionnaire items will be rated on seven-point scales with appropriately worded anchors, usually 'strongly disagree' to 'strongly agree'. The questionnaire will be piloted for clarity and acceptability to both clinical and non-clinical staff.

Individuals' cognitions about work characteristics (both in general and in relation to diabetes care provision) will be measured using Karasek's job decision latitude scale [45] and job demands scale [49], and Siegrist's effort-reward imbalance measure [50]. Cognitions about the team will be measured using the shortened version of the original team climate inventory [46,51]. Cognitions about the organisation will be measured using the organizational justice evaluation scale [52,53].

### Dependent variables

#### Data relating to the process of care, markers of biochemical control, and QOF scores

Data for dependent variables will be collected from four sources. We will study both the performance of clinical behaviours (measuring HbA1c, BP) and the associated biochemical/physiological measurement (level of HbA1c; level of BP) accepting that there will be variability in the latter measures reflecting patient physiology and behaviour. However, these are the sort of criteria by which quality of care is judged. We will also measure clinician self-report and patient report of clinician behaviour. All dependent variables will relate to the same twelve-month period (12 months from the completion of the initial theory-based questionnaire).

#### Data held within practice computers on the performance of clinical behaviours and measures of physiology

These data will be collected in two ways. First, for all registered patients with diabetes we will collect data on the total number of patients with diabetes in the practice and the number who have had: a foot check; BP, HbA1c, cholesterol, and weight measured; level of systolic and diastolic BP, level of HbA1c, level of cholesterol, and body mass index; diabetes-related medication (hypoglycaemic drugs, lipid-lowering drugs, weight-reducing drugs); advice about self-monitoring and education.

Second, we will collect QOF scores for diabetes and for additional items that reflect aspects of good organisation and that, on the basis of QOF scores, can be expected to discriminate between practices (*e.g*., Records 18 (The practice has up-to-date clinical summaries in at least 80 percent of patient records); Education 6 (The practice conducts an annual review of patient complaints and suggestions to ascertain general learning points which are shared with the team); Med 9 (A medication review is recorded in the notes in the preceding 15 months for all patients being prescribed repeat medicines.). These data are available as categorical variables [54].

#### Clinician self-reported behaviour

Two measures of clinicians' self-reported behaviour that can be regarded as proxies for actual behaviour will be included in the initial theory-based questionnaire – behavioural simulation and behavioural intention. Behavioural simulation will be measured using the method we have used previously [27]. From literature and expert consensus we will identify elements reported to influence management of the clinical conditions. From this, clinical scenarios will be constructed describing patients presenting in primary care. Respondents will be asked to make decisions on the management of the patients described. Behavioural intention will be measured by three items worded in a standard manner (*e.g*., I intend to control the BP of my patients with diabetes rated on a seven-point scale from 'strongly disagree' to 'strongly agree'). Responses to the three items will be summed [55]. A third measure of self-reported behaviour will be used. After a period of 12 months a questionnaire will be sent to clinical staff asking them about their behaviour in relation to the same six aspects of care over the preceding twelve months.

#### Patient-report of clinicians' behaviour

Three dependent variables representing proxy measures of clinicians' behaviour will be generated from a questionnaire survey of a random sample of 100 patients with diabetes from each practice. A self-administered questionnaire will be developed that will ask patients if, over the previous 12 months, they were: offered advice about self-management, and if so, what this advice entailed; received advice about losing or controlling their weight, and if so, what this advice entailed; received or were offered education, and if so what this education entailed.

The content of the questionnaire will include selected sections of the questionnaire used by the healthcare commission in their 2006 national survey of people with diabetes (*e.g*., self-management and knowledge, education and training). This will provide data that are interpretable in the context of a pre-existing national survey. These questions will be supplemented with specific knowledge questions developed in collaboration with our study team and our local diabetes UK voluntary support group. Patients will also be asked about their uptake of the advice given, and what they did or currently do in terms of acting on it. The final questionnaire will be piloted with patients from the voluntary group.

### Administration of data collection

Data relating to practice organisational structure will be collected by telephone interview with the study contact at each recruited practice. This will also identify recipients of the questionnaires and will include members of the administrative staff (*e.g*., receptionist staff) and clinicians who are attached rather than employed (district nurses), although we are aware that a previous study found it was not possible to gather usable data from this latter group [3].

Questionnaires will be delivered to the practices where the research nurse (or other nominated study contact) will be responsible for their distribution, collection, and return. Reminders will be sent to practices at two-week intervals.

For clinical data held within practice computers, the research nurses will run computer queries to extract data on the total number of patients with diabetes in the practice and the number who have had the relevant clinical actions performed.

Data relating to clinician self-reported behaviour will be measured in the postal questionnaire (above) and, in addition, after a period of 12 months, a second, short questionnaire will be sent to clinical staff asking them about their behaviour in relation to the same six aspects of care over the preceding twelve months.

For the patient-reported data, questionnaires will be sent out to the 100 randomly selected patients from each practice by the nominated study contact at each participating practice. To protect patient confidentiality, a reply paid envelope, addressed to the Institute of Health and Society at Newcastle, will be provided for each patient to return their completed questionnaire directly to the research team. No reminders will be used.

### Sample size

For the practice-held, QOF, and clinician self-reported data, the analysis program 'G Power' [56] (using the method described by Cohen [57]) has been used to investigate the sample size required for testing alternative regression models. In this approach, the effect size is defined as the proportion of variance accounted for by a set of predictor variables (anticipated to be 14) relative to the residual variance proportion. In this context, effect sizes of 0.02, 0.15 and 0.35 are considered to be 'small', 'medium' and 'large', respectively. For the analysis of practice level variables (practice behaviour and the aggregated intention and behavioural simulation variables) with 100 practices we will have 80% power to detect an effect size of 0.21 assuming a type one error rate of 5% and 14 predictor variables. Assuming a (worst case) response rate of 75%, we will have data from all relevant staff (100 practices, four GPs, three practice nurses, one practice manager, two receptionists) the estimated sample size will be around 750 which, assuming a type one error rate of 5% and 14 predictor variables will give us 80% power to detect an effect size of 0.03. Thus, in the analyses of individual level data we will have 80% power to detect effect sizes in the small to medium range, and at the practice level we will have 80% power to detect effect sizes in the medium to large range.

For the patient-reported data, making the conservative assumption that the estimated proportion is around 50% if we sample 25 patients per practice, the standard error associated with our estimate for each practice is 10%. We will approach 100 randomly selected patients per practice (10,000 patients in total), allowing for a 25% response rate to achieve a final sample size of 25 per practice.

### Statistical analysis

The study will generate data on:

1. Individuals' cognitions about clinical behaviours, clinical conditions, work, team and organisational setting.

2. Organisational structure and function.

3. Clinical behaviours relating to teams of clinicians.

4. Biochemical and physiological measurements that are the consequence of the clinical behaviours, but include consequences of patient physiology and behaviour.

The analysis will explore the relationships between the predictor variables (one and two) and the dependent variables of respondents' intentions to perform behaviours, respondent's behavioural simulation, (both from survey instruments), and the clinical behaviours (three) as well as biochemical and physiological measures (four).

Where appropriate, data will be analysed as the standardised (by list size) number of patients who have had the investigation/procedure of interest performed; standardised number of patients who have their level of the investigation/procedure of interest within the accepted target range. From the patient survey we will calculate the proportion of patients in each practice who have a particular attribute (*e.g*., have been given education or training in self-monitoring).

Where appropriate (*i.e*., more than two items per measure), the reliability of the measures will be assessed prior to analysis using Cronbach's alpha to assess internal reliability and confirmatory factor analysis to identify and discard redundant items. The construct validity of the measures will be assessed prior to analysis by examining correlations between predictor variables that are expected to be similar (convergent validity) and dissimilar (discriminant validity).

We will examine mediating and moderating effects of the predictor variables on the dependent variables. In principle, we will be dealing mainly with dependent variables measured at the level of the practice and predictor variables measured at either the level of the practice or the level of the individual. The theoretical models will be tested using standard multiple regression analysis and structural equation modelling. For predictor variables that are measured at the level of the individual, we will produce a summary statistic for the practice. We will use the practice mean and, in the multiple regression analysis, will weight by practice size. We will explore other summary statistics with weightings reflecting the relevant roles and responsibilities of the respondents.

In addition to the main study analysis, the patient survey will be reported in its own right, including a comparison with the healthcare commission data (available from their website), a comparison of the level of agreement between patients' and health professionals' perceptions and a comparison of patient perceptions and physiological measures of diabetes control.

## Ethics committee review

The study has been approved by Newcastle and North Tyneside Research Ethics Committee Two (REC Ref Number 07/H0907/102).

## Competing interests

The authors declare that they have no competing interests.

## Authors' contributions

The study was conceived by MPE, JMG, JJF and MJ. It was designed by MPE, GH, MJ, JJF, NS, MH, ME and JMG. The patient survey was conceived by MPE, SH, MJ, JJF and was designed by MPE, SH, JJF, MJ, GH, NS, MH. Writing of the manuscript was led by SH and MPE. All authors commented on all drafts and approved the final version.
